# Metabolic Features of Women With Polycystic Ovary Syndrome in Latin America: A Systematic Review

**DOI:** 10.3389/fendo.2021.759835

**Published:** 2021-10-19

**Authors:** Lucas Bandeira Marchesan, Ramon Bossardi Ramos, Poli Mara Spritzer

**Affiliations:** ^1^ Gynecological Endocrinology Unit, Division of Endocrinology, Hospital de Clínicas de Porto Alegre, Porto Alegre, Brazil; ^2^ Post-graduate Program in Endocrinology, Medicine School, Universidade Federal do Rio Grande do Sul, Porto Alegre, Brazil; ^3^ Department of Physiology, Universidade Federal do Rio Grande do Sul, Porto Alegre, Brazil

**Keywords:** obesity, metabolic syndrome, insulin resistance, PCOS (polycystic ovary syndrome), Latin America

## Abstract

**Background:**

Polycystic ovary syndrome (PCOS) is an endocrine disorder that commonly affects women of childbearing age and has been associated with metabolic and reproductive abnormalities. Only a few studies have investigated metabolic traits in women with PCOS in Latin America. Therefore, we conducted a systematic review to provide an overview of the available evidence on the metabolic profile of Latin American women with PCOS.

**Methods:**

We searched PubMed, Cochrane Central Register of Controlled Trials, and Embase databases for cross-sectional, case-control, or cohort studies focusing on populations of countries in South and Central America and Mexico, published until October 31, 2019. We selected studies that reported the diagnostic criteria for PCOS. In the absence of a control group, we included studies if they reported relevant metabolic data.

**Results:**

The initial search yielded 4878 records, of which 41 studies were included in the systematic review. Sample sizes ranged from 10 to 288 in PCOS groups and from 10 to 1500 in control groups. The prevalence of phenotypes A and B (classic PCOS) ranged from 65.8% to 87.5% as reported in studies from Argentina, Brazil, and Chile. Metabolic syndrome ranged from 33.3% to 44.0% for phenotype A, from 15.0% to 58.0% for phenotype B, from 11.9% to 36.0% for phenotype C, and from 14.2% to 66.0% for phenotype D. Women with PCOS had higher body mass index, waist circumference, blood pressure, glucose, and homeostasis model assessment index as well as a more adverse lipid profile than those without PCOS.

**Conclusions:**

Evidence from the present systematic review suggests that anthropometric and metabolic profiles are worse in women with PCOS who live in different Latin American countries than in women without PCOS living in the same region. Additional studies assessing metabolic comorbidities, such as diabetes, and distinct PCOS phenotypes in different Latin American countries are warranted and may produce invaluable information for primary and secondary prevention of PCOS in the region. This systematic review was registered with PROSPERO under number CRD42016038537.

**Systematic Review Registration:**

PROSPERO, identifier CRD42016038537.

## Introduction

Polycystic ovary syndrome (PCOS) is an endocrine condition that commonly affects women of childbearing age. The etiology of PCOS is uncertain, but the available evidence strongly suggests that its onset is triggered by environmental, genetic, and behavioral factors that interact in a complex manner (1–3).

Obesity affects the majority of women with PCOS, placing them at increased risk for impaired glucose tolerance, metabolic abnormalities, and type 2 diabetes (4–7), and possibly for cardiovascular and cerebrovascular events and venous thromboembolism (2, 8–11). Insulin resistance with compensatory hyperinsulinemia affects approximately 65% to 70% of women with PCOS (12). An estimated 30%–40% of patients with PCOS have impaired glucose tolerance, and 7.5%–10% have type 2 diabetes (13–15). While the prevalence of insulin resistance is high in both lean and obese women with PCOS (16), the presence of obesity may exacerbate the development of metabolic comorbidities and cardiovascular risk factors (17–19).

Many studies have investigated the prevalence of PCOS and related metabolic abnormalities in different continents. A recent meta-analysis showed a lower prevalence of PCOS in Chinese women than in white (Caucasian), Middle Eastern (Iranian and Turkish), and black (African American and Afro-Brazilian) women (20). However, the prevalence of PCOS and metabolic profile has not yet been described in several ethnic groups, especially in Latin American populations (6, 17, 21, 22), except for a recent meta-analysis of metabolic disturbances in Brazilian women with PCOS (23). Therefore, we conducted the present systematic review to provide an overview of the available evidence on the metabolic profile of Latin American women with PCOS, as well as the frequency of different PCOS phenotypes in this population.

## Methods

### Search Strategy and Study Selection

A systematic review was designed and described in agreement with the Preferred Reporting Items for Systematic Reviews and Meta-analyses (PRISMA) guidelines. This systematic review was registered with PROSPERO under number CRD42016038537. We searched PubMed, Cochrane Central Register of Controlled Trials, and Embase databases for cohort, case-control, cross-sectional, and prevalence studies with populations of South and Central America and Mexico, published until October 31, 2019. We set no language or publication date restrictions. To identify eligible studies, we used medical subject headings (MeSH) for PubMed and Ovid Tree terms for Embase. We used the following search strategy for PubMed, with equivalent terms being used in the other databases: “Polycystic Ovary Syndrome” [MeSH] OR “Ovary Syndrome, Polycystic” OR “Syndrome, Polycystic Ovary” OR “PCOS” OR “Polycystic Ovarian Syndrome” OR “Ovarian Syndrome, Polycystic” OR “Polycystic Ovary Syndrome 1” AND “Body Mass Index” [MeSH] OR “Metabolic Syndrome” OR “Glucose Intolerance” [MeSH] OR “Intolerance, Glucose” OR “Intolerances, Glucose” OR “Diabetes Mellitus, Type 2” [MeSH]. We performed additional searches in review articles and research articles focusing on PCOS.

We selected only studies that clearly defined the diagnostic criteria for PCOS and that included at least one of the following variables in the analysis: waist circumference (WC), body mass index (BMI), glucose levels, lipid profile, homeostasis model assessment of insulin resistance (HOMA-IR), blood pressure, diabetes mellitus, metabolic syndrome (MetS), PCOS prevalence, and milder PCOS phenotypes. Eligibility assessment was done by screening the titles and abstracts of all articles selected, and when abstracts did not provide the necessary information, the full text of the article was reviewed. This was performed independently, in a standardized manner, by two investigators (RBR and LBM). Disagreements between reviewers were resolved with discussion. If a consensus was not reached, a third investigator (PMS) was consulted. When articles had missing information, we contacted the authors for further information. In the case of duplicate data that had been published more than once, we opted to include the most complete study. In addition, the reference lists of all articles fulfilling the eligibility criteria were hand searched to identify other essential citations.

### Data Extraction and Quality Control Assessment

Data were individually extracted by two researchers (LBM and RBR), and agreement was pursued in all extracted items. When an agreement could not be achieved, data extraction discrepancies were resolved by referring to the original publication or by consulting a third reviewer (PMS). Data extracted from each study included: name of the authors, country, publication year, type of study, characteristics of the population, diagnostic criteria, total sample size, and outcomes of interest in the PCOS and control groups. We assessed the quality of observational studies included in this systematic review using the Newcastle-Ottawa Scale (NOS). The NOS uses a “star system” to judge the quality of the studies in three broad perspectives: selection of the study groups, comparability of the groups, and ascertainment of the outcome of interest. Each item contains a sequence of alternative questions to be answered by the investigators. Then, a star rating system allows the semi-quantitative analysis of article quality. No statistical quantitative meta-analysis was performed due to study heterogeneity.

## Results

### Flowchart of Study Selection

**Figure 1:**
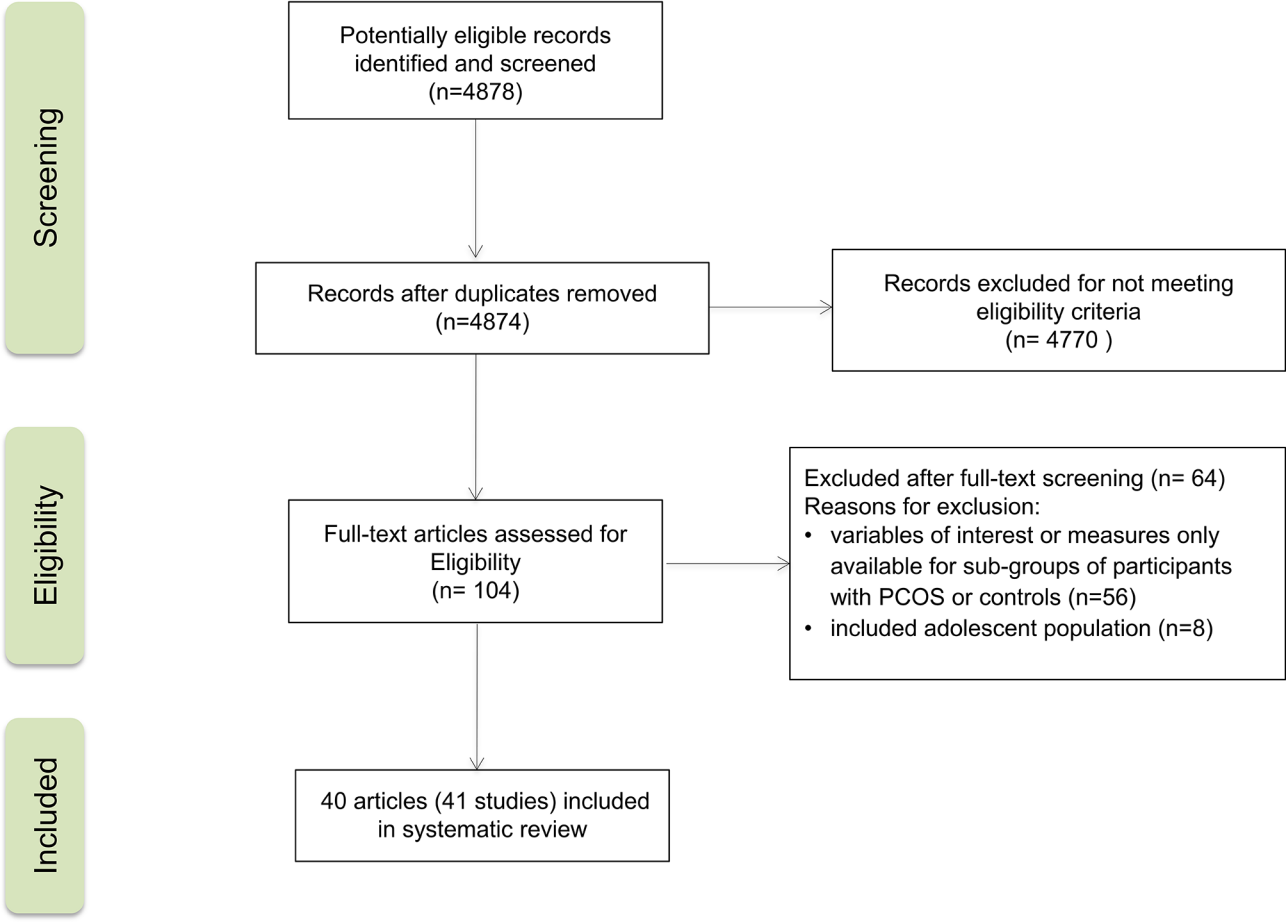
PRISMA flow diagram of the study selection process.

**Figure 1** provides a flowchart summarizing the study selection process. The initial search yielded 4878 records. Of these, 41 studies from 40 reports were included in the systematic review. All of them were observational studies: 24 cross-sectional studies, 16 case-control studies, and one cohort study. Publication years ranged from 2004 to 2019. PCOS group size ranged from 10 to 288 participants, and control group size ranged from 10 to 1500 participants. Age ranged from 20.6 to 31.1 years for women with PCOS and from 22.7 to 34.5 years for non-PCOS controls.

### Characteristics of Included Studies

**Table 1** presents the characteristics of studies, which included populations from Argentina (n=3) (24–26), Brazil (n=27) (27–53), Chile (n=8) (26, 54–60), Venezuela (n=2) (62, 63), and Mexico (n=1) (61). Most studies used the Rotterdam criteria to diagnose PCOS, except for one study conducted in Argentina (25), one in Brazil (27), and three in Chile (54, 58, 60), all of which used the National Institutes of Health (NIH) criteria. The two studies from Venezuela (62, 63) used criteria defined by the authors. Sixteen studies had no control group for comparison (24, 26, 27, 35, 38, 39, 43–45, 47, 51, 53, 57, 59, 63), and six had BMI-matched controls without PCOS for comparison (28, 29, 34, 40, 50, 55). NOS score was 7-9 in 33 studies and ≤ 6 in 7 (**Table 2**).

**Table 1:** Characteristics of the studies from Latin America included in the systematic review about women with PCOS.

Country	Study, Year	PCOS criteria	Type of studies	PCOS		Control group		BMI-matched
				N	Age	N	Age	
Argentina	Belli, et al., 2004 (24)	Rotterdam	Cross-sectional	24	23.7 ± 6.4	–	–	
	Tellechea, et al., 2013 (25)	NIH	Case-control	165	26.4 ± 0.5	121	30.7 ± 0.78	
	de Guevara, et al., 2014 (26)	Rotterdam	Cross-sectional	206	26.0 (18–39)	–	–	
	Santana, LF, et al., 2004 (27)	NIH	Cohort	21	27.2 ± 5.02	–	–	
Brazil	Costa LO, et al., 2008 (28)	Rotterdam	Cross-sectional	57	25.5 ± 5.3	37	26.6 ± 5.4	yes
	Wiltgen D, et al., 2009 (29)	Rotterdam	Case-control	51	20.6 ± 5.1	44	28.9 ± 5.6*	yes
	Cerqueira J, et al., 2010 (30)	Rotterdam	Cross-sectional	56	26.2 ± 6.0	54	27.7 ± 6.1	
	Wiltgen D, et al., 2010^a^ (31)	Rotterdam	Case-control	195	22.3 ± 6.7	25	29.7 ± 4.29*	
	Azevedo MF, et al., 2011 (32)	Rotterdam	Cross-sectional	113	26.2 ± 4.3	242	26.8 ± 5.0	
	Melo AS, et al., 2011^b^ (33)	Rotterdam	Cross-sectional	132	26.6 ± 5.1	146	28.9 ± 0.5	
	Rocha MP, et al., 2011 (34)	Rotterdam	Case-control	142	25.1 ± 5.4	31	27.5 ± 4	yes
	Costa, et al., 2012 (35)	Rotterdam	Cross-sectional	113	27.2 ± 4.5	–	–	
	Gabrielli L, et al., 2012 (36)	Rotterdam	Cross-sectional	73	28.4 ± 6.5	725	31.0 ± 7.3*	
	Kogure GS, et al., 2012 (37)	Rotterdam	Case-control	20	27.8 ± 5.0	19	27.9 ± 5.2	
	Pedroso DCC, et al., 2012 (38)	Rotterdam	Cross-sectional	105	29 ± 4.4	–	–	
	Pontes AG et al., 2012 (39)	Rotterdam	Cross-sectional	189	24.9 ± 5.2	–	–	
	Lauria PB, et al., 2013 (40)	Rotterdam	Case-control	40	29 (25–34)	36	30 (15–43)	yes
	Oliveira RS, et al., 2013^c^ (41)	Rotterdam	Case-control	42	27.4 ± 5.5	18	31.4 ± 6.1	
	Radavelli-Bagatini S, et al., 2013 (42)	Rotterdam	Case-control	80	21.3 ± 0.6	1500	22.7 ± 0.4	
	Avila MA, et al., 2014 (43)	Rotterdam	Cross-sectional	100	25.7 ± 4.9	–	–	
	de Medeiros SF, et al., 2014 (44)	Rotterdam	Cross-sectional	288	26.9 ± 5.5	–	–	
	Maciel, et al., 2014 (45)	Rotterdam	Cross-sectional	97	24.9 ± 5.1	–	–	
	Ramos RB, et al., 2015 (46)	Rotterdam	Case-control	199	22 ± 6	99	25 ± 7	
	Soares, et al., 2016 (47)	Rotterdam	Cross-sectional	22	26 ± 6.0	–	–	
	Carvalho, et al., 2017 (48)	Rotterdam	Case-control	86	31.1 ± 4.92	86	29.0 ± 7.04	
	Graff, et al., 2017 (49)	Rotterdam	Case-control	84	23.5 ± 6.3	54	26.2 ± 6.5	
	Simões, et al., 2017 (50)	Rotterdam	Case-control	10	29.6 ± 1.2	10	28.6 ± 2.0	yes
	Wanderley, et al, 2018 (51)	Rotterdam	Cross-sectional	83	28.79 ± 5.85	–	–	
	Xavier, LB, et al., 2018 (52)	Rotterdam	Case-control	97	30.5 ± 5.1	99	29.8 ± 7.1	
	Tavares A, et al., 2019 (53)	Rotterdam	Cross-sectional	111	18-39	–	–	
Chile	Bravo, et al., 2005 (54)	NIH	Case-control	106	23.5± 5.19	82	25.1± 5.64	
	Cerda C, et al., 2007 (55)	Rotterdam	Case-control	41	24.6± 7.2	31	27.9± 6.9	yes
	Codner, et al., 2007 (56)	Rotterdam	Cross-sectional	20	24.5 ± 5	35	26.4 ± 7.2	
	Vigil, et al., 2007 (57)	Rotterdam	Cross-sectional	69	26.01 ± 0.76	–	–	
	Márquez, et al., 2008 (58)	NIH	Cross-sectional	50	28.8 ± 8.2	70	28.6 ± 8.6	
	de Guevara, et al., 2014 (26)	Rotterdam	Cross-sectional	220	26.0 (18–39)	–	–	
	Echiburú, et al., 2014^d^ (59)	Rotterdam	Cross-sectional	60	22.3 ± 5.3	–	–	
	Echiburú, et al., 2016^e^ (60)	NIH	Cross-sectional	43	27 (23–30)	38	29 (20–30)	
Mexico	Moran C, et al., 2010 (61)	Rotterdam	Cross-sectional	10	28.9 ± 2	140	34.5 ± 7	
Venezuela	Roa Barrios, et al., 2009 (62)	Other^f^	Case-control	62	23.9 ± 0.6	48	25.4 ± 0.7	
	Quintero-Castillo, et al., 2010 (63)	Other^f^	Cross-sectional	65	23.2 ± 4.92	–	–	

^a^ data are from A plus B PCOS phenotypes vs controls; ^b^ data are from A PCOS phenotype vs controls; ^c^ women included in the control group had similar complaints as the ones from the PCOS group, but did not meet the diagnostic criteria; ^d^ data are from baseline and regarding the phenotype A only; ^e^ data shown from the early reproductive age group (18–34 years); ^f^PCOS diagnosis defined by the authors; * p < 0.05 between the groups.

**Table 2:** Newcastle-Ottawa quality (NOS) assessment scale for studies included in the systematic review.

Author	Year	Selection	Comparability	Exposure/Outcome
Belli, et al.	2004	****	*	**
Tellechea, et al.	2013	****	**	***
de Guevara, et al.	2014	****	**	***
Santana, LF, et al.	2004	***	*	**
Costa LO, et al.	2008	***	*	***
Wiltgen D, et al.	2009	****	**	***
Cerqueira J, et al	2010	**	*	***
Wiltgen D, et al.	2010	****	**	***
Azevedo MF, et al.	2011	****	**	***
Melo AS, et al.	2011	****	**	***
Rocha MP, et al.	2011	****	**	***
Costa, et al.	2012	***	*	**
Gabrielli L, et al.	2012	***	**	***
Kogure GS, et al.	2012	****	**	***
Pedroso DCC, et al.	2012	***	*	**
Pontes AG et al.	2012	****	*	**
Lauria PB, et al.	2013	***	*	***
Oliveira RS, et al.	2013	***	*	***
Radavelli-Bagatini S, et al.	2013	****	*	***
Avila MA, et al.	2014	****	*	***
de Medeiros SF, et al.	2014	****	*	***
Maciel, et al.	2014	****	*	***
Ramos RB, et al.	2015	****	**	***
Soares, et al.	2016	****	*	***
Carvalho, et al.	2017	****	**	***
Graff, et al.	2017	****	*	***
Simões, et al.	2017	****	**	***
Wanderley, et al.	2018	****	*	**
Xavier, LB, et al.	2018	****	**	***
Tavares A, et al.	2019	***	*	**
Bravo, et al.	2005	****	**	***
Cerda C, et al.	2007	****	**	***
Codner, et al.	2007	****	**	***
Vigil, et al.	2007	**	*	*
Márquez, et al.	2008	****	**	***
Echiburú, et al.	2014	**	*	*
Echiburú, et al	2016	****	**	***
Moran C, et al.	2010	***	**	***
Roa Barrios, et al.	2009	***	**	***
Quintero-Castillo, et al.	2010	****	*	***

Quality of selection for case/control (minimum 1 – maximum 4 stars); Comparability (minimum 0 – maximum 2 stars); Exposure (minimum 1 – maximum 3 stars).

Quality of selection adapted for cross-sectional/cohort studies (minimum 0 – maximum 5 stars); Comparability (minimum 0 – maximum 2 stars); outcome (minimum 0 – maximum 3 stars).

### Qualitative Data

**Figure 2:**
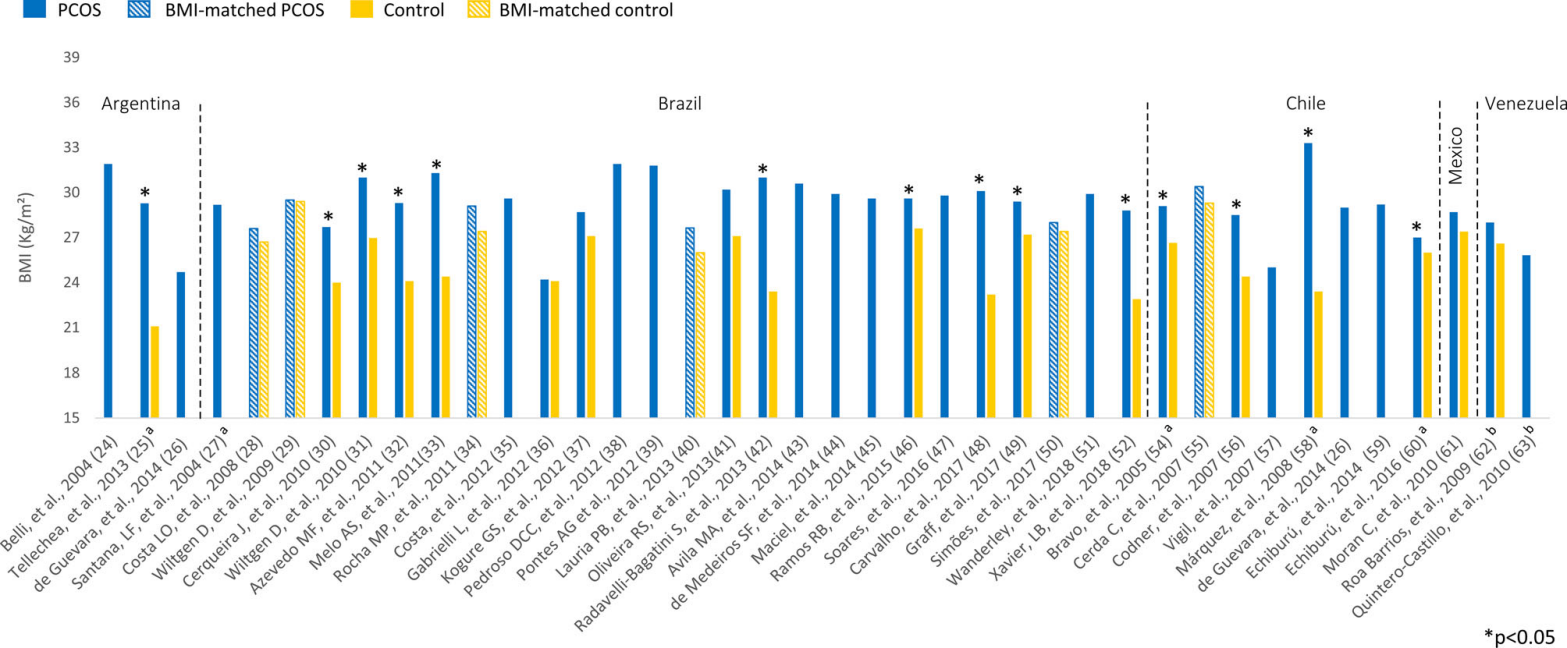
BMI (kg/m²) among Latin American women with PCOS and controls. Mean values. The “x” axis shows the name of studies and reference numbers (refer to the text). ^a^ PCOS diagnosis according to NIH criteria; ^b^ PCOS diagnosis defined by the authors.

**Figure 3:**
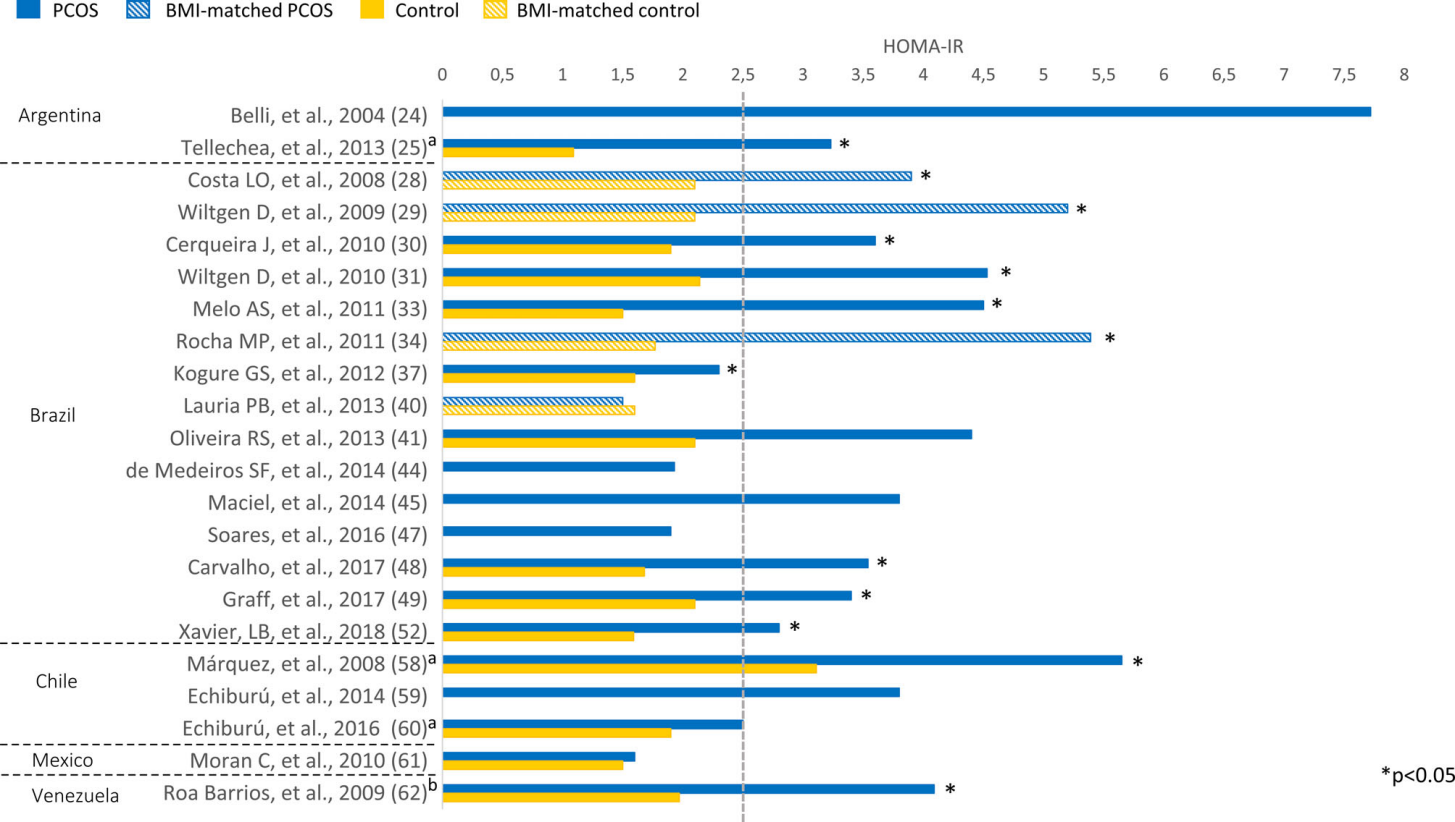
HOMA-IR among Latin American women with PCOS and controls. Mean values. The “x” axis shows the name of studies and reference numbers (refer to the text). ^a^ PCOS diagnosis according to NIH criteria; ^b^ PCOS diagnosis defined by the authors.

Overweight (BMI 25-29.9 kg/m²) or obesity (BMI ≥ 30 kg/m²) was prevalent among Latin American women with PCOS (**Figure 2**). BMI ranged from 24.2 to 33.3 kg/m² in women with PCOS. Most studies comparing women with PCOS *versus* BMI-unmatched controls showed higher BMI in PCOS groups (25, 30–33, 42, 46, 48, 49, 52, 54, 56, 58, 60). Several studies also assessed HOMA-IR, a marker of insulin resistance, in women with PCOS (**Figure 3**). HOMA-IR was > 2.5 in women with PCOS in 16 studies, six of them with obese participants (24, 31, 33, 41, 48, 58) and the others with overweight women (25, 28–30, 34, 45, 49, 52, 59, 62). In six studies HOMA-IR was ≤ 2.5 (37, 40, 44, 47, 60, 61), all of them with overweight participants. Seventeen studies compared HOMA-IR between women with PCOS and non-PCOS controls. HOMA-IR was higher in women with PCOS than in controls in 13 studies, 10 BMI-unmatched (25, 30, 31, 33, 37, 48, 49, 52, 58, 62) and 3 BMI-matched (28, 29, 34). While HOMA-IR was > 2.5 in most studies from Argentina, Brazil, Chile, and Venezuela, it was < 2.5 in the only included study from Mexico (61) (**Figure 3**).

**Figure 4:**
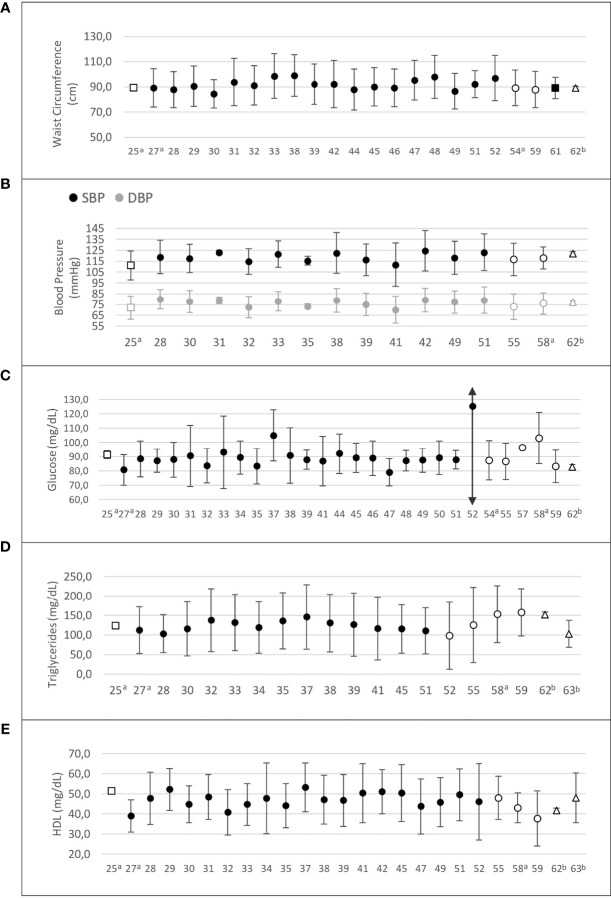
Risk factors composing the metabolic syndrome in Latin American women with PCOS. **(A)** Waist circumference (cm); **(B)** systolic and diastolic blood pressure (mm Hg); **(C)** fasting glucose (mg/dL); **(D)** triglycerides (mg/dL); **(E)** HDL-cholesterol (mg/dL). Values are expressed as mean and standard deviation. The “x” axis shows the reference number of studies (refer to the text). □ Argentina; •Brazil; ○ Chile; ∆ Venezuela. ^a^ PCOS diagnosis according to NIH criteria; ^b^ PCOS diagnosis defined by the authors.

**Figure 4** summarizes the variation of MetS components among studies of women with PCOS in Latin American countries. Central obesity (WC ≥ 88 cm) was prevalent among women with PCOS, who had higher WC values than controls in 13 of the 20 studies that reported this information (**Supplementary Table 1**).

Fifteen studies reported blood pressure data for PCOS and control groups (25, 28, 30–33, 40–42, 46, 49, 55, 58, 60, 62) (**Figure 4**). In nine of these studies, women with PCOS had higher systolic (SBP) and/or diastolic blood pressure (DBP) than controls (28, 30–33, 42, 46, 49, 58). One study evaluated blood pressure as a MetS component and found a higher prevalence of this criterion in the PCOS group, considering a 130/85 mm Hg cutoff point (35.1% *vs.* 7.1%, p=0.005, PCOS *vs.* controls) (46). Another study found higher SBP and DBP only in late reproductive-age (35–40 years) women with PCOS (60). Blood pressure levels were homogeneously distributed across countries. However, in all four studies from Chile, where these data were available, the mean SBP and DBP would be classified as “normal” according to the 2017 American College of Cardiology/American Heart Association (ACC/AHA) definition of high blood pressure (64) (**Supplementary Table 1**).

Fasting glucose was measured in 31 studies (25, 27–35, 37–41, 44–52, 54, 55, 57–60, 62). Glucose levels ranged from 79 to 125.2 mg/dL in women with PCOS. In six of 21 studies (25, 30, 32, 33, 54, 58), women with PCOS had higher glucose levels than controls (**Supplementary Table 2**). Mean fasting glucose was homogeneously distributed across countries, and in most of them mean glucose levels were within the reference range. However, in two studies from Brazil (37, 52) and in one from Chile (58), mean fasting glucose was within the prediabetes range in patients with PCOS (**Figure 4**).

Regarding lipid profile, 26 studies showed triglyceride levels ranging from 81 to 157.8 mg/dL (**Supplementary Table 2**). Triglyceride levels were higher in women with PCOS than in controls in 11 of 17 studies (25, 29–33, 37, 52, 58, 60, 62). One BMI-matched study (29) also found higher triglyceride levels in the PCOS group. Whereas Brazilian and Argentinian studies showed mean triglyceride levels within the reference range, two studies from Chile (58, 59) and one from Venezuela (62) reported mean triglyceride levels > 150 mg/dL in patients with PCOS (**Figure 4**).

Twenty-seven studies assessed high-density lipoprotein cholesterol (HDL-C), and 18 of them compared HDL-C levels between PCOS and control groups (25, 28–34, 37, 40–42, 46, 49, 55, 58, 60, 62). In 10 of these studies, HDL-C was significantly lower in women with PCOS than in controls (25, 28, 30, 32–34, 42, 46, 49, 58). In the remaining studies, HDL-C levels did not differ between PCOS and control groups (**Supplementary Table 2**). In most studies, patients with PCOS had HDL-C < 50 mg/dL (27, 28, 30–35, 38–40, 47, 49, 51, 52, 55, 58–60, 62, 63). One study of women with PCOS conducted in Argentina reported HDL-C > 50 mg/dL (25), and studies of Brazilian women with PCOS showed variable HDL-C results, but mostly below the cutoff point of 50 mg/dL (27, 28, 30–35, 38–40, 47, 49, 51, 52). Studies from Chile and Venezuela reported mean HDL-C levels below this cutoff point (55, 58–60, 62, 63) (**Figure 4**).

Low-density lipoprotein cholesterol (LDL-C) levels ranged from 88.6 to 127.3 mg/dL in women with PCOS in 24 studies. Six of 15 studies comparing data between women with PCOS and controls reported higher LDL-C levels for PCOS (28, 29, 40, 42, 52, 58). LDL-C was within the reference range in control groups (**Supplementary Table 2**).

In 25 studies, mean total cholesterol levels ranged from 167 to 209.7 mg/dL in PCOS groups. Eight of 17 studies showed higher total cholesterol levels for women with PCOS than controls (25, 29, 30, 40, 42, 52, 58, 62) (**Supplementary Table 2**).

The prevalence of PCOS was estimated in only two studies. One study was conducted in Mexico (61) with a convenience sample of 150 female Mexican volunteers aged 20 to 45 years, and the authors found a prevalence of 6.6% (95% confidence interval, 2.3%–10.9%) according to the Rotterdam criteria. The other study was conducted in the city of Salvador, Brazil (36), and estimated a prevalence of 8.5% using the Rotterdam criteria in a probability sample of 859 women aged 18 to 45 years.

Six studies reported prevalence data on PCOS phenotypes and on MetS stratified by phenotype (26, 31, 33, 53, 59) for Brazilian, Chilean, and Argentinian populations. Phenotypes A+B were more prevalent in all studies, with rates ranging from 65.8% to 87.5%. The prevalence of MetS ranged from 33.3% to 44.0% for phenotype A, from 15.0% to 58.0% for phenotype B, from 11.9% to 36.0% for phenotype C, and from 14.2% to 66.0% for phenotype D (**Table 3**).

**Table 3:** Prevalence of PCOS phenotypes and of Metabolic syndrome in the studies included in the systematic review.

Study, year	Country	PCOS criteria	Type of study	N PCOS phenotypesA+B/C/D	Age range PCOS (ys) phenotypes	Prevalence PCOS phenotypes (%)	Prevalence Met S (%)
**de Guevara, et al., 2014** (26)	Argentina	Rotterdam	Cross-sectional	144/41/21	18 - 39	A+B: 69.9 C:19.9 D:10.2	A: 36.2 B: 15 C:12.2 D:14.2
**Wiltgen D, et al., 2010** (31)	Southern Brazil	Rotterdam	Cross-sectional	195/45/-	A+B:22.3 ± 6.7 C: 25.89-7.56 D:-	A+B: 81 C: 19	A+B:31.3 C:11.9 D:-
**Melo AS, et al., 2011** (33)	Southeastern Brazil	Rotterdam	Cross-sectional	150/25/51	A: 26.6 ± 5.1 B: 26.2 ± 5.7 C: 27 ± 4.5 D: 25.9 ± 5.3	A+B:66.4 C:11 D:22.6	A: 45 B:39 C:36 D:33
**Tavares A, et al., 2019** (53)	Northeast Brazil	Rotterdam	Cross-sectional	73/16/22	18-39	A+B: 65.8 C: 14.4 D: 19.8	A:33.3 B: 30.8 C: 12.5 D: 36.4
**de Guevara, et al., 2014** (26)	Chile	Rotterdam	Cross-sectional	181/36/3	18 - 39	A+B:82.5 C:16.5 D:1	A: 44 B:58 C: 30 D:66
**Echiburú B, et al., 2014^a^** (59)	Chile	Rotterdam	Cross-sectional	77/9/2	A: 22.3 ± 5.3 B: 24.9 ± 7.3 C: 25.7 ± 5.7 D:24.5 ± 14.8	A+B: 87.5 C: 10.2 D: 2.3	A+B: NA C: NA D: NA

^a^Data from baseline.

## Discussion

PCOS is a complex disorder affecting metabolic and reproductive functions. This systematic review, which included 24 cross-sectional studies, 16 case-control studies, and one cohort study conducted in Latin America, found that women with PCOS had a more adverse metabolic profile than non-PCOS controls across different countries. In most studies, BMI was within the overweight or obesity range for women with PCOS, reinforcing its contribution to the disease phenotype. In addition, MetS components, such as central obesity (measured by WC), low HDL-C, and hypertension, were prevalent in women with PCOS from different Latin American countries.

Although efforts have long been made to assess the impact of different sociocultural and ethnic backgrounds on PCOS-related metabolic abnormalities, few data are available for Latin America. This region is known to have populations of different ancestry. In Brazil, pooled ancestry contributions have been listed as 0.62 European, 0.21 African, and 0.17 Amerindian (65), whereas Pacific Latin American countries are predominantly Amerindian. Argentina and Chile are particular cases that show similar European and Amerindian ancestry contributions but lower African ancestry contribution compared with Brazil (65, 66). It is reasonable to assume that different genetic backgrounds may influence the phenotypic heterogeneity of PCOS, but evidence from the present systematic review rather suggests that Latin American countries are similar in terms of metabolic traits. This information may be potentially useful to public health systems in developing PCOS prevention programs and policies.

Metabolic abnormalities are considered common in women with PCOS, especially those linked to the MetS cluster, as shown in this study. However, controversy exists as to whether these features are directly related to PCOS itself or dependent on obesity—mainly on abdominal adiposity, a well-known cardiometabolic risk factor (7, 67, 68). In this respect, the finding of decreased insulin sensitivity in Latin American women with PCOS, as opposed to controls, is in line with current evidence from other regions (6, 15) and has been associated with low-grade chronic inflammation, linked to increasing BMI (68, 69). Besides, in meta-analyses of different populations, women with PCOS were more likely to have MetS (4, 17, 70). However, these studies provide relatively few data from Latin American populations. Insulin resistance may actually drive most of the alterations observed in PCOS, even in nonobese women. While not universally present in patients with PCOS, the presence of insulin resistance has been considered an intrinsic factor independent of obesity (71, 72). Recently, we have also observed an association of insulin resistance with hypertension, regardless of BMI, in Brazilian women with PCOS, with hypertension being associated with other MetS components (18). Data from the present systematic review add support to this notion by showing that Latin American women with PCOS had higher HOMA-IR than controls in most studies.

Although patients with PCOS consistently show a more unfavorable metabolic profile than controls in different regions of the world, there are discrepancies between PCOS populations. In China, the prevalence of MetS in PCOS ranged from 18.2% in community-dwelling patients in one study (73) to 53.3% in women older than 40 years in another study (74). In a prospective cohort of 479 women with PCOS from Vietnam (Southeast Asia), patients were lean, had no increase in metabolic disease and Rotterdam phenotype D was the most prevalent (67.6%) (75). Current evidence also indicates a lower prevalence of hyperandrogenemia in women with PCOS from Asian countries (76). In Latin America, we found a predominance of Rotterdam phenotypes A and B, similar to what has been reported in most of the available studies across the world (76). A recent meta-analysis reported that, compared with controls, patients with PCOS from North America had a higher risk of MetS than those from Asia and Europe (17). Likewise, in the present systematic review, we also found a high prevalence of MetS in Latin American women with PCOS. In addition to the ethnic composition of the population, dietary habits may also influence the expression of metabolic traits in different populations. Indeed, adherence to the Mediterranean diet (77) or a low-glycemic-index diet (78) has been associated with a better metabolic profile in PCOS. Regarding the dietary pattern in Latin America, the Latin American Study of Nutrition and Health (ELANS) (79) reported low consumption of vegetables, nuts, whole grains, fish, and yogurt according to the recommendations of the World Health Organization. This may explain, at least in part, the similarities in the adverse metabolic profile between Latin American countries and other countries with high consumption of processed foods (80).

Despite the paucity of research undertaken to date, the results of the present systematic review provide a broad overview of the evidence on metabolic and anthropometric parameters in women with PCOS living in Latin American countries. The comprehensive search strategy can be seen as a strength of this study, as it covered the major electronic databases in order not to miss any relevant articles and included an active search for publications without language restrictions. Limitations include the relatively few studies found despite the vast size of the region, possible heterogeneity between studies, small sample sizes, and a lack of studies in some countries of the region, which hindered a proper comparison between women with PCOS from different Latin American countries. Nevertheless, no similar analysis has yet been undertaken. The present study is the first to provide evidence that allows us to characterize the metabolic profile of women with PCOS from an array of sociocultural and ethnic backgrounds in Latin American countries.

## Conclusions

The results of the present systematic review suggest that anthropometric and metabolic profiles are worse in women with PCOS who live in different Latin American countries than in women without PCOS living in the same region. These findings are similar to those from North America but differ from the milder phenotype seen in Asia and Europe. Further studies assessing the prevalence of cardiometabolic comorbidities, such as diabetes and hypertension, in Latin American countries are needed, which could positively impact the prevention and management strategies for PCOS.

## Data Availability Statement

The original contributions presented in the study are included in the article/**Supplementary Material**. Further inquiries can be directed to the corresponding author.

## Author Contributions

LM contributed to study design, was involved with data collection and analysis, drafted the article and final review. RR contributed to study design, was involved with data collection and analysis, drafted the article and final review. PS was involved in the conception and design of the study, data collection and analysis, drafted the article and final review. All authors contributed to the article and approved the submitted version.

## Funding

This work was funded by the Conselho Nacional de Desenvolvimento Científico e Tecnológico (grant number INCT/CNPq 465482/2014–7) and Fundação de Amparo à Pesquisa do Estado do Rio Grande do Sul (grant number INCT/FAPERGS: 17/2551–0000519-8). The funding source had no role in the collection, analysis, interpretation of data and in the writing of the report or in the decision to submit the article for publication.

## Conflict of Interest

The authors declare that the research was conducted in the absence of any commercial or financial relationships that could be construed as a potential conflict of interest.

## Publisher’s Note

All claims expressed in this article are solely those of the authors and do not necessarily represent those of their affiliated organizations, or those of the publisher, the editors and the reviewers. Any product that may be evaluated in this article, or claim that may be made by its manufacturer, is not guaranteed or endorsed by the publisher.
