# Supplementary material for: Metabolic Features of Women With Polycystic Ovary Syndrome in Latin America: A Systematic Review
Source: Front Endocrinol (Lausanne). 2021 Oct 19;12:759835. doi: 10.3389/fendo.2021.759835 (PMC8562723; doi:10.3389/fendo.2021.759835)
Supplement: Supplementary file 1 [file Table_1.docx]

Supplementary Table 1. BMI, waist circumference and blood pressure in Latin American women with PCOS in the studies included in the systematic review

| **Country** | | **Study, year** | **BMI (kg/m^2^)**  **(PCOS/Controls)** | **Waist circumference (cm) (PCOS/controls)** | **Blood pressure (mmHg)**  **(PCOS/controls)** |
| --- | --- | --- | --- | --- | --- |
| Argentina |  | Belli, et al., 2004 (24) | 31.9 ± 7.8/ - | Waist to hip ratio  0.86±0.08/- | NA |
|  |  | Tellechea, et al., 2013^†^  (25) | 29.28±0.63/21.08±0.18* | 89.3±1.6/71.0±0.6* | SBP: 111.2±1.7/109.7±2.3  DBP: 72.2±1.38/72.7±0.91 |
|  |  | de Guevara, et al ., 2014 (26) | 24.7 (18 – 35)/ - | 88(68-120)/- | SBP: 120(100-150)/-  DBP: 80(60-95)/- |
|  | | Santana, LF, et al., 2004^†^ (27) | 29.18±7.78/- | 89.36±15.23/- | NA |
| Brazil | | Costa LO, et al., 2008 (28) | 27.6±5.8/26.7±4.9 | 87.8±14.3/83.6±10.1 | SBP: 118.9±15.2/113.8±10.9  DBP: 79.9±8.9/73.4±10.2* |
|  |  | Wiltgen D, et al., 2009 (29) | 29.5 ± 7.5/29.4 ±5.4 | 90.6±16.1/ 85.5±11.6* | NA |
|  |  | Cerqueira J, et al., 2010 (30) | 27.7 ±5.4/ 24 ±4.2* | 84.5±11.3/ 78.9±10.0* | SBP:117.5±11.9/104.0±10.3*  DBP:77.7±9.8/68.7±8.1* |
|  |  | Wiltgen D, et al., 2010^a^ (31) | 31±7.98/ 26.97 ±3.6* | 93.79±18.81/ 79.83± 8.37* | SBP: 123.1±16.9/115.2±9.5*  DBP: 78.9±12.3/ 73.6±8.3 |
|  |  | Azevedo MF, et al., 2011 (32) | 29.3 ±6.7/ 24.1±4.4* | 91.2±15.7/77.1±9.6* | SBP: 114.8±13.3/111.5±10.7*  DBP: 72.6±10.7/72.1±10.3 |
|  |  | Melo AS, et al., 2011^b^ (33) | 31.3±8.7/24.4 ± 4.9* | 98.6±17.7/84.8±12.4 | SBP: 121.5±15.2/111.5±10.8*  DBP: 78±10.4/71.7±8.2* |
|  |  | Rocha MP, et al., 2011 (34) | 29.1±6.17/27.4±6.9 | NA | NA |
|  |  | Costa, et al., 2012 (35) | 29.6±6.6/- | NA | SBP:115.5±13.0/-  DBP:73.3±10.3/- |
|  |  | Gabrielli L, et al., 2012 (36) | 24.2 (17.7–30.7)/ 24.1 (18.1–30.1) | 74 (56-92)/75 (60-90) | SBP: 119 (99-139)/122 (102-142)  DBP: 74 (60-88)/ 74 (59.5-89.5) |
|  |  | Kogure GS, et al., 2012 (37) | 28.7±4.4/27.1±5.1 | NA | NA |
|  |  | Pedroso DCC, et al., 2012 (38) | 31.9±8.2/- | 99±16.6/- | SBP:122.5±18.7/-  DBP:79±11/- |
|  |  | Pontes AG et al., 2012 (39) | 31.8 ±7.6/- | 92.2±16.0/- | SBP:116.3±14.4/-  DBP:75.1±10.4/- |
|  |  | Lauria PB, et al., 2013 (40) | 27.64 ±5.43/25.99±5.51 | 91 (83-101)/94 (83-103) | SBP: 120 (110-120)/120 (110-20)  DBP: 80 (70-80)/ 80 (70-80) |
|  |  | Oliveira RS, et al., 2013^c^ (41) | 30.2 ±6.5/27.1 ±6.2 | NA | SBP: 111.8±12.0 / 107.3±15.0  DBP: 70.2±9.0/71±13.5 |
|  |  | Radavelli-Bagatini S, et al., 2013 (42) | 31.0 ± 7.9/23.4 ±4.6* | 92.2±18.8/74.5±10.2* | SBP: 124.6±19.9/111.5±13.0*  DBP: 79.2±12.5/71.8±10.6* |
|  |  | Avila MA, et al., 2014 (43) | 30.6 ±9.3/- | NA | NA |
|  |  | De Medeiros SF, et al., 2014 (44) | 29.9 ±7.0 /- | 88.0±16.3/- | NA |
|  |  | Maciel, et al., 2014 (45) | 29.6± 6.9 /- | 90.1±15.2/- | NA |
|  |  | Ramos RB, et al., 2015 (46) | 29.6 ±6.4/ 27.6 ±6.0* | 89.2±15.0/78.1±11.5* | NA |
|  |  | Soares, et al., 2016 (47) | 29.8±6.1/- | 95.4±15.8/- | NA |
|  |  | Carvalho, et al., 2017 (48) | 30.1±5.4/ 23.2 ± 4.23* | 98± 17.0/71.5± 16.0* | NA |
|  |  | Graff, et al., 2017 (49) | 29.4± 6.4/27.2± 5.8* | 86.6±14.1/83.6±12.3 | SBP:118.2±13.0/112.4±11.1*  DBP:77.4±9.9/72.8±10.0* |
|  |  | Simões, et al., 2017 (50) | 28.0±2.4/27.4± 2.4 | NA | NA |
|  |  | Wanderley, et al., 2018 (51) | 29.9± 5.28/ - | 92.15±10.72/ - | SBP: 123.15±18.38/-  DBP: 79.13±11.00/- |
|  |  | Xavier, LB, et al., 2018 (52) | 28.8 ±8.1/ 22.9 ±5.9* | 97.0±18.0/ 82.4± 20.0* | NA |
| Chile | | Bravo, et al., 2005^†^ (54) | 29.1±6.08/26.65±5.34* | 89.2±14.1/83.6±12.9* | NA |
|  |  | Cerda C, et al., 2007 (55) | 30.4 ±7.1/29.3 ±5.3 | Waist to hip ratio  0.87±0.08/ 0.86±0.08 | SBP:116.66±14.79/116±16.53  DBP:73±11.85/73.1±13.08 |
|  |  | Codner, et al., 2007 (56) | 28.5± 6.6/24.4±3.3* | Waist to hip ratio  0.86±0.1/0.77±0.0* | NA |
|  |  | Vigil, et al., 2007 (57) | 25.01±0.54/- | NA | NA |
|  |  | Márquez, et al., 2008^†^ (58) | 33.3 ± 8.1/23.4±2.7* | NA | SBP:118±10/110±11*  DBP:76±10/70±10 |
|  |  | de Guevara, et al ., 2014 (26) | 29.0 (18.2 – 35)/ - | 90(59-126)/- | SBP:112(100-160)/-  DBP: 70(50-100)/- |
|  |  | Echiburú, et al., 2014^d^ (59) | 29.2±5.9/- | 87.9±14.5/- | NA |
|  |  | Echiburú, et al., 2016 ^e†^ (60) | 27.0 (24.7-29.7)/26.0(23.1-27.9)* | 82(77-92)/73(68-79)* | SBP:110(100-120)/110(100-120)  DBP: 70(60-80)/70(60-70) |
| Mexico | | Moran C, et al., 2010 (61) | 28.7 ±3.4/ 27.4 ±4.6 | 89.1±8.4/86.2±11.0 | NA |
| Venezuela | | Roa Barrios, et al., 2009^‡^ (62) | 28±0.6/26.6±0.8 | 89.16±1.36/80.64±1.46* | SBP:122±2/119±2  DBP:77±3/76±2 |
|  |  | Quintero-Castillo, et al., 2010^‡^ (63) | 25.82±3.44/- | Waist to hip ratio  0.83±0.08/- | NA |

^a^ data are from A plus B PCOS phenotypes *vs* controls; ^b^ data are from A PCOS phenotype *vs* controls; ^c^ women included in the control group had similar complaints as the ones from the PCOS group, but did not fulfill the diagnostic criteria; ^d^ data are from baseline and regarding phenotype A only; ^e^ data refer to early reproductive age group (18–34 years); * p< 0.05 between the groups. Continuous metabolic variables were not available for Tavares et al., 2019 (53).
^†^PCOS diagnosis according to NIH criteria; ^‡^ PCOS diagnosis defined by the authors.
