# Supplementary material for: Metabolic Features of Women With Polycystic Ovary Syndrome in Latin America: A Systematic Review
Source: Front Endocrinol (Lausanne). 2021 Oct 19;12:759835. doi: 10.3389/fendo.2021.759835 (PMC8562723; doi:10.3389/fendo.2021.759835)
Supplement: Supplementary file 2 [file Table_2.docx]

Supplementary Table 2. Glucose and lipid profile in Latin American women with PCOS in the studies included in the systematic review

| **Country** | **Study, year** | **Total cholesterol (mg/dL)** | | **HDL (mg/dL)** | | **LDL (mg/dL)** | | **Triglycerides (mg/dL)** | | **Glucose (mg/dL)** | |  |
| --- | --- | --- | --- | --- | --- | --- | --- | --- | --- | --- | --- | --- |
| Argentina | Belli, et al., 2004 (24) | | NA | | NA | | NA | | NA | | NA | |
|  | Tellechea, et al., 2013^†^  (25) | | 188.7±3.48/164.35±3.09* | | 51.43±1.55/56.84±1.55* | | NA | | 124±7.09/67.32±2.66* | | 91.26±2.7/81±0.72* | |
|  | de Guevara, et al ., 2014 (26) | | NA | | NA | | NA | | NA | | NA | |
| Brazil | Santana, LF, et al., 2004^†^  (27) | 181.7±38.09/- | | 39±8.05/- | | 119.8±33.06/- | | 111.8±59.82/- | | 80.7±10.7/- | |  |
|  | Costa LO, et al., 2008 (28) | 169±24.2/159±32.2 | | 47.7±13/ 59.4±10.3* | | 99.2±25.1/85.3±27.7* | | 103.1±48.7/94.3±59.9 | | 88.4 ±12.6/87.7 ±7.5 | |  |
|  | Wiltgen D, et al., 2009 (29) | 187.64±42.85/ 162.54±30.88* | | 52.12±10.42/50.96±11.58 | | 118.91±37.45/ 94.98±26.64* | | 97.4 (68.2-131)/64.6 (47.8-107.1)* | | 87.1 ±8.2/88.5 ±7.5 | |  |
|  | Cerqueira J, et al., 2010 (30) | 176.9±31.4/159.0±29.3* | | 44.8±9.2/ 54.5±16.7* | | 88.6±35.6/82.6±29.1 | | 115.6±69.0/83.1±38.0* | | 87.9 ±12.3/84.3 ±7.3* | |  |
|  | Wiltgen D, et al., 2010^a^ (31) | 182.4 ±43.79/ 165.28±36. 82 | | 48.43±11.18/54.96±13.71 | | 110.15± 37.28/ 95.5±31.49 | | 99.5 (67.25–142)/ 60 (42 – 93)* | | 90.52±21.42/ 88.67±8.19 | |  |
|  | Azevedo MF, et al., 2011 (32) | 186.50±42.0/176.9±46.0 | | 40.8±11.3/53.8±18.9* | | NA | | 138.0±80.9/100.5±52.8 * | | 83.6 ±12.1/76.5 ±11.2* | |  |
|  | Melo AS, et al., 2011^b^ (33) | 184±40.4/190.5±32.7 | | 44.7±10.4/54.7±10.7* | | 112.3±35.7/109.0±28.0 | | 132±72.4/84±38.7* | | 93.1 ±25.4/82.6 ±8.8* | |  |
|  | Rocha MP, et al., 2011 (34) | 180.88±30.31/165.34±15.49 | | 47.74±17.56/58.32±7.91* | | 111.0±25.36/101.54±22.49 | | 118.97±66.03/117.32±35.81 | | 89.3 ±11.7/85.2 ±7.3 | |  |
|  | Costa, et al., 2012 (35) | 188.7±41.8/- | | 44.1 ±11.0/- | | 120.3±41.3/ - | | 136.1±72.2/ - | | 83.3±12.3/- | |  |
|  | Gabrielli L, et al., 2012 (36) | NA | | NA | | NA | | NA | | NA | |  |
|  | Kogure GS, et al., 2012 (37) | 209.7±48.1/211.5±46.1 | | 53.2±12.2/58.7±10.9 | | 127.3±35.6/131.6±37.2 | | 146.2±82.8/105.5±66.9* | | 104.8 ±17.9/103.3 ±19.6 | |  |
|  | Pedroso DCC, et al., 2012 (38) | 187.8±39.1/- | | 47.1±12.2/- | | 113.6±33.7/- | | 130.6±74.1/- | | 90.8±19.4/- | |  |
|  | Pontes AG et al., 2012 (39) | 182.6±34.8/- | | 46.7±12.9/- | | 111.9±29.7/- | | 126.7±80.9/- | | 87.8±6.8/- | |  |
|  | Lauria PB, et al., 2013 (40) | 169 (142-184)/142 (124-168)* | | 43 (34-50)/39 (33-44) | | 107 (89-126)/87 (68-111)* | | 81 (65-108)/71 (58-94) | | 84 (77–93)/86 (81–92) | |  |
|  | Oliveira RS, et al., 2013^c^ (41) | NA | | 50.3±14.8/57.5±13.5 | | NA | | 116.8±80.6/116.5±66.0 | | 86.8 ±17.4/81.8 ±8.4 | |  |
|  | Radavelli-Bagatini S, et al., 2013 (42) | 186±45/164±35* | | 51±11/59±14* | | 114±38/81±28* | | NA | | NA | |  |
|  | Avila MA, et al., 2014 (43) | NA | | NA | | NA | | NA | | NA | |  |
|  | De Medeiros SF, et al., 2014 (44) | NA | | NA | | NA | | NA | | 92±14/- | |  |
|  | Maciel, et al., 2014 (45) | 171 ±31.6/ - | | 50.4±14.1/ - | | 98.6 ±26.1/ - | | 115.2±62.1/ - | | 89.1±10.3/ - | |  |
|  | Ramos RB, et al., 2015 (46) | NA | | NA | | NA | | NA | | 88.8 ± 12.2/88.4±7.5 | |  |
|  | Soares, et al., 2016 (47) | NA | | 43.7±13.7/- | | 89(70-104)/- | | 123(63-164)/- | | 79.0±9.6/- | |  |
|  | Carvalho, et al., 2017 (48) | NA | | NA | | NA | | NA | | 87.10±7.26/ 84.75± 10.42 | |  |
|  | Graff, et al., 2017 (49) | 173.7±34.3/174.0±30.4 | | 45.8±12.2/50.6±11.0* | | 106.5±27.4/105.4±24.3 | | 88.0(61.0-135.5)/77.5(53.5-102.8) | | 87.4±8.4/86.8±7.9 | |  |
|  | Simões, et al., 2017 (50) | NA | | NA | | NA | | NA | | 89.2±11.9/81.8±8.6 | |  |
|  | Wanderley, et al., 2018 (51) | 183.07±34.88/ - | | 49.47±12.91/ - | | 117.16±32.74/ - | | 110.6±59.09/ - | | 87.74±6.52/ - | |  |
|  | Xavier, LB, et al., 2018 (52) | 189.9±36.2/175.7±32.4* | | 46±19/54± 19* | | 114.6±31.8/99.6±28* | | 98±86/82± 39* | | 125.2±201.8/104.2±128.7 | |  |
| Chile | Bravo, et al., 2005^†^  (54) | NA | | NA | | NA | | NA | | 87.4±13.8/80.1±12.24* | |  |
|  | Cerda C, et al., 2007 (55) | 185.96±43.31/179.09.9±44.5 | | 47.97±10.82/47.29±11.88 | | 106.7±34.63/119.85±33.03 | | 125.57±96.61/127.62±62.23 | | 86.7± 12.8/82.0±8.7 | |  |
|  | Codner, et al., 2007 (56) | NA | | NA | | NA | | NA | | NA | |  |
|  | Vigil, et al., 2007 (57) | NA | | NA | | NA | | NA | | 96.38±1.12/- | |  |
|  | Márquez, et al., 2008^†^  (58) | 201±36/181±30* | | 43±7.5/53±11.8* | | 127±41/107±32* | | 153±73/81±42* | | 103±18/90±14* | |  |
|  | de Guevara, et al ., 2014 (26) | NA | | NA | | NA | | NA | | NA | |  |
|  | Echiburú, et al., 2014^d^ (59) | 179.4±58.2/- | | 37.7±13.8/- | | 111.5±55.4/- | | 157.8±60.9/- | | 83.2±11.4/- | |  |
|  | Echiburú, et al., 2016^e†^ (60) | 167(143-198)/163(150-183) | | 41.5(34-48.9)/41.9 (37.6–46.7) | | 96(77-125)/106(91-118) | | 145(102-177)/92(71-127)* | | 82(75-90)/85(76-98) | |  |
| Mexico | Moran C, et al., 2010 (61) | NA | | NA | | NA | | NA | | NA | |  |
| Venezuela | Roa Barrios, et al., 2009^‡^ (62) | 188.9±6.2/170.5±5.3* | | 41.7±1.2/43.3±1.6 | | 117±5.5/105.7±5.1 | | 151.2±7/106.7±5.1* | | 82.6±1.7/82.5±1.3 | |  |
|  | Quintero-Castillo, et al., 2010^‡^ (63) | 169.16±31.35/- | | 47.95±12.47/- | | 102.83±23.5/- | | 102.67±34.4/- | | NA | |  |

^a^ data are from A plus B PCOS phenotypes *vs* controls; ^b^ data are from A PCOS phenotype *vs* controls; ^c^ women included in the control group had similar complaints as the ones from the PCOS group, but did not fulfill the diagnostic criteria; ^d^ Data are from baseline and regarding phenotype A only; ^e^ data refer to early reproductive age group (18–34 years); * p< 0.05 between the groups. Continuous metabolic variables were not available from Tavares et al., 2019 (53). ^†^PCOS diagnosis according to NIH criteria; ^‡^ PCOS diagnosis defined by the authors.
